# Monitoring the Shelf Life of Refined Vegetable Oils under Market Storage Conditions—A Kinetic Chemofoodmetric Approach

**DOI:** 10.3390/molecules27196508

**Published:** 2022-10-02

**Authors:** Sandra Martín-Torres, Juan Antonio Tello-Jiménez, Rafael López-Blanco, Antonio González-Casado, Luis Cuadros-Rodríguez

**Affiliations:** 1 Department of Analytical Chemistry, Faculty of Science, University of Granada, C/Fuentenueva s/n, E-18071 Granada, Spain; 2 Laboratory ‘J.A. Tello’, S.L., Industrial Park “Los Olivares”, C/La Iruela No 8, E-23009 Jaén, Spain

**Keywords:** multivariate deterioration monitoring, multiparametric-kinetic studies, chemometrics, shelf life, quality and stability indices, refined vegetable oil

## Abstract

Most physicochemical and sensory properties of edible vegetable oils are not stable over time. One of the main causes of quality depletion of vegetable oils is oxidation, which influences sensory acceptability and nutritional value, and could even lead to toxic compounds. That negative influence affects international refined oil prices and the variety of its culinary applications. Modelling quality depletion of vegetable oils and establishing the shelf life, generally accepted as the time until rancidity becomes evident, already remains a challenge for the industry. Hence, this paper will show a promising chemofoodmetric methodology, as an easy and straightforward tool to estimate the current shelf-life of refined vegetable oils, based on a comprehensive characterisation of quality depletion-related changes over storage time under real market conditions. The methodology for building a multivariate kinetic ageing-based model is described, taking into account all time-related physicochemical parameters and chemometric processing tools. From a particular ageing state, multiparametric models are able to reliably infer the expected storage time for each vegetable oil so that it remains consistent with acceptability requirements. The results of the study pointed out the accuracy of multivariate shelf-life modelling with regard to univariate modelling. Discrepancies were found in the oxidation rates of oils extracted from different plant seeds.

## 1. Introduction 

Vegetable oils are important constituents of food and are essential components of the human diet. Vegetable oils are obtained by mechanical expelling or solvent extraction of oleaginous seeds (soybeans, rapeseed, sunflower, etc.) or oleaginous fruit, such as palm, avocado, or olive [1]. Among them, consumption of refined vegetable oils has been growing strongly in recent years. Attractive international edible vegetable oil prices (cheaper than virgin oils such as olive oil), together with improved producer gross profit margins, increase in cultivation area, as well as multiple industrial and culinary applications make palm, sunflower, soybean, rapeseed, canola, corn and grapeseed oils the most consumed edible vegetable oils worldwide [2].

Knowledge of information related to the potential or expected shelf life (oxidative stability) of culinary refined vegetable oils is therefore of great importance. Palatability, nutritional quality, and, in some instances, toxicity and physicochemical changes need to be checked before the oils are marketed, incorporated into food products, and/or used by the consumers as salad oils/dressings. Oxidative stability is defined as the resistance to oxidation and can be expressed as the period of time necessary for the critical point of oxidation, whether it is a sensorial change or a sudden acceleration of the oxidative process [3]. Because a large portion of phospholipids is removed by degumming in the refining process of vegetable oils, oxidation of refined edible vegetable oils primarily involves the oxidation of triacylglycerols. Lipid oxidation is much more complex than the simplistic radical chain reactions normally presented [4]. During oxidation, usually promoted by initiators such as light and temperature, unsaturated fatty chains derive mainly in the formation of hydroperoxides, primary oxidation products. Hydroperoxides, tasteless and odourless, are generally unstable and react to form secondary lipid oxidation products such as aldehydes, ketones, alcohols, organic acids, epoxides, and hydrocarbons. These secondary oxidation products contribute off-flavour notes that significantly impact the sensory quality of edible vegetable oils/fats and foods containing oils/fats. In general, this negative characteristic of the product is known as rancidity, and it is the main cause of consumer rejection. Additionally, oxidation products, together with free radicals, may have an adverse effect on the human body or even be toxic after intake [5,6,7]. 

One question that persists in lipid oxidation analytical control is which oxidation products are the most effective to monitor. Most food companies define shelf life in their own ways and follow their own criteria for defining a quality informative index for considering a product acceptable or unacceptable. That fact implies that the same vegetable oil bottled by two different companies could have different expiration dates. One criterion for bottled vegetable oil is that the consumer should not be able to smell any oxidized or rancid aroma during handling and cooking, so that only sensory perception is monitored. However, do all consumers perceive the same threshold? Another common approach is that vegetable oils cannot exceed a peroxide value (PV) regulated by definitive guidelines [8]. However, PV only provides information about the primary oxidation compound. It does not take into account the secondary oxidation compounds, which are actually responsible for the off-odours. Another common approach is to estimate the shelf life of edible vegetal oils in a relatively short period of time by subjecting the sample to an accelerated oxidation test under standardized conditions and choosing a suitable endpoint to determine appropriate levels of oxidative deterioration. An example is the Rancimat test, based on automatically determining the time before the maximum change of rate of oxidation by measuring the increase in conductivity of deionized water caused by dry air bubbled through a heated sample, which takes the resulting volatile acids into a separate recipient with the deionized water. However, estimations from these methods do not often correlate with real shelf life under real storage conditions due to the widely divergent kinetics of lipid oxidation at unusual conditions employed in the accelerated tests [9].

In this sense, the authors propose for the first time the use of the term chemofoodmetrics to refer to the science that relates measurements of chemical or physicochemical parameters to the quality features of a food by applying mathematical or statistical methods, which have been proved to lead to advances in the understanding of complex reactions that take place in foodstuffs. Among them, multiparametric kinetic studies and chemometric modelling approaches by applying specific data mining methods have been proven more generically applicable than single-parameter models to assess shelf life, and could be more easily applied to other products or processes [10,11,12]. This paper is aimed at estimating the shelf life of most commonly consumed refined edible vegetable oils in Spain when oxidised under standard storage conditions relying on multiparametric modelling. The basics of the applied methodology are subsequently described.

## 2. Materials and Methods

### 2.1. Samples

A total of 60 refined vegetable oil samples, bought in grocery supermarkets (Granada and Jaen, Spain), were used in this study, distributed as follows: 29 samples of sunflower oil; 1 sample of grapeseed oil; 1 sample of rapeseed oil; 4 samples of corn oil; 4 samples of vegetable oil blend constituted by different (not stated) seeds; 17 samples of olive oil (marketed as a blend of virgin and refined olive oil); and 4 samples of pomace oil. The requirement was established that the vegetable oils should have been bottled for the shortest possible time so that only those vegetable oils with at least 20 months remaining until the ‘best before’ consumption date (shown in the bottle) were selected. 

### 2.2. Storage

Twelve batches were considered, consisting of the 60 different refined vegetable oils, which were packaged in transparent food-grade PET 60 mL bottles (labelled A to L) and were stored in a temperature-controlled room (20 ± 5 °C) exposed to 12 h of cold white LED light (6200 K), simulating standard market store conditions for 24 months (actual shelf life testing). The comprehensive control of storage conditions, i.e., temperature and exposure to oxygen and light, is a critical step for carrying out a shelf-life study. The first lot (A), considered as two months of ageing, was analysed at the beginning of the study. After 2 months of storage, lot (B) was analysed. Consecutively, every two months, an aliquot of each vegetable oil sample was analysed (from lot (C) to lot (L) after 24 months of ageing) for determining physicochemical and sensory (volatile-based) parameters.

### 2.3. Physico-Chemical Testing and Analytical Equipment

Characteristic primary and secondary oxidation-related analytical parameters were carefully chosen to be monitored as feasible quality indicators to monitor oxidative changes as a function of time. The fatty acid profiles of all the oils were analysed by GC-FID chromatography at the beginning of the study with the purpose of ensuring identity. Results are shown in the supplementary material (Table S1). 

The refractive index was determined according to the ISO 280 standard [13] using an Abbe refractometer ORT1RS (Kern & Sohn, Albstadt, Germany). Spectroscopic UV absorptivity values, K_232,_ K_270,_ and ∆K were determined in compliance with COI/T.20/Doc. No 19 standard [14] on a Genesys 10SUV-Vis spectrophotometer (Thermo Scientific, Waltham, USA). The oxidative stability was performed on Metrohm 892 Professional Rancimat at 120 °C, as described in the ISO 6886 standard [15]. In addition, both peroxide and anisidine values were determined according to the recognised methods described in COI/T.20/Doc. No 35 [16] and ISO 6885 standard [17], respectively. For the last one, an Agilent 8453 UV-Vis spectrophotometer (Agilent Technologies, Santa Clara, CA, USA) was used. 

Total α-, β-, γ- y δ-tocopherols were quantified according to the ISO 9936 standard [18]. The analytical determination was performed on an Agilent 1100 Series liquid chromatograph (Agilent Technologies, Santa Clara, CA, USA) equipped with a G1321A fluorescence detector using an Ultrabase Sil column. 

Authorised antioxidant additives, such as butylated hydroxytoluene (BHT), butylated hidroxyanisole (BHA), *tert*-butyl-hydroquinone (TBHQ), and propylgallate (PG), were quantified by high-performance liquid chromatography (Agilent 1260 Series, Agilent Technologies, Santa Clara, CA, USA) and successive UV molecular absorption measurements using a diode-array detection (DAD) system following the principles outlined in the IUPAC method [19] with slight modifications of the chromatography conditions. A Zorbax Eclipse Plus C18 4.6 × 50 mm, 5 μm column was used. A flow rate of 1.2 mL/min and a column temperature of 30 °C were set. An amount of 20 μL of the sample was injected; 5% (*v/v*) solution of acetic acid in water (A) and 5% (*v/v*) solution of acetic acid in acetonitrile (B) were used as mobile phases, and the following gradient was applied: from 30% to 100% B from 0 to 7 min, hold at 100% B for 10 min, return to 30% B in 1 min and re-equilibrate for 4 min. 

For each vegetable oil, volatile compounds were firstly fractioned by headspace solid-phase microextraction (HS-SPME) and then a representative chromatographic fingerprint was acquired using a gas chromatographer (Varian 3800, Palo Alto, CA, USA) equipped with a flame ionization detector (Varian 450 GC, Palo Alto, CA, USA). A more detailed explanation of both sample preparation and chromatographic conditions is given in a previous paper from our laboratory [20]. After the acquisition, volatile fingerprints were exported from the instrument software, embedded in a data vector, and further pre-processed: (i) selection of a region of interest; (ii) alignment using an average chromatogram; and (iii) normalization of intensities with respect to the internal standard. Each data vector containing the related chromatographic fingerprint of a refined vegetable oil sample consisted of 8391 elements, each of which is a single variable indicating a normalised signal intensity. When the fingerprinting methodology is applied, the compounds referred to are neither identified nor quantified in the conventional way. It is therefore necessary to apply a variable selection step in order to retain those that best define the evolution of vegetable oil ageing. 

### 2.4. Kinetic Modelling and Shelf Life Estimation

As argued previously, modelling food quality features means modelling changes, as the quality of a food nearly always changes over time. Food quality modelling is therefore almost synonymous with kinetic modelling. The consequence is that certain differential equations frequently form the basis for mathematical models; these can sometimes be solved analytically but if not, it is relatively easy nowadays to solve them numerically with the available software, or even using conventional spreadsheets [21]. 

Two chemometric multivariate tools are used for kinetic modelling and shelf life prediction, respectively, when large data sets are available: principal component analysis (PCA) and partial least-squares linear regression (PLS). 

PCA is an unsupervised exploratory method based on the assumption that high variability is synonymous with a large amount of information. For this reason, PCA algorithms search for the maximum variance direction, in the multidimensional space of the original data. The maximum variance direction represents the first principal component (PC). It is usually employed for feature and noise reduction purposes and constitutes the basis for other more complex pattern recognition techniques [22]. Because of that, the PCA model was built in order to detect the presence or absence of outlier samples, and PC scores were used to model the kinetic of refined oil oxidation.

PLS is probably the most widely used multivariate regression technique and represents a better solution to both the problems of variable number and intercorrelation. The latent variables (LVs) are directions in the space of the predictors (supervised method). In particular, the first LV is the direction characterized by the maximum covariance with the selected response variable. PLS regression was used to evaluate the importance of individual variables in the overall quality depletion model using a loading plot of the variable’s importance in projection (VIP) scores in a PLS model. LV scores were used to obtain a multivariate equation of oil quality depletion and, therefore, to evaluate shelf life.

One of the critical steps in assessing shelf life and distinguishing from a stability study is to properly establish an acceptability limit from which shelf life values could be calculated. When a multivariate shelf life model is conducted, the acceptability limit consists of a data vector which includes the limit values, usually referring to legal or regulatory requirements, of all the concerned physicochemical features that show change over time, and gives rise to a single scalar parameter, Q_c_, which is traduced to shelf life time:

$$Q_{C}=\;\max\;(Q_{A}\cdot \;L)$$

where **Q_A_** is the autoscaled acceptability limit vector and **L** the loading matrix of the time-related latent variable of the model. Note that the term in parentheses is a scalar product of two vectors and hence results in a scalar. Q_C_ is then interpolated into the shelf life model equation to obtain the cut-off criteria, t_C_, or the shelf life time.

As far as refined vegetable oils are concerned, there are several regulatory limits depending on whether they are refined vegetable oils from olives, i.e., olive and pomace oils [23], or other vegetable oils [24]. Because of that, samples were modelled separately, divided into two groups: (i) olive-pomace refined oils, and (ii) other refined seed (sunflower, grapeseed, rapeseed, corn, and blended) vegetable oils. The results of both groups of samples are discussed below. 

## 3. Result and Discussion

### 3.1. Selecting of Influential Volatile Variables

In order to carry out a previous selection of the volatile variables influencing the ageing of vegetable oils being considered, all volatile fingerprints were integrated into a first data matrix. Then, a preliminary PLS regression model against storage time was built and the ‘variable importance in projection’ (VIP) coefficients were calculated. VIP formulation is a combined measure of how much a variable contributes to describing the two data sets; the dependent variable (y-variable) and the independent variable (x-variable). A variable with a VIP coefficient close to or greater than one can be considered relevant in a given model. Variables with VIP coefficients lower than one are less important and might be good candidates for exclusion from the model. Variables (linked to retention times) showing an absolute VIP coefficient exceeding the threshold value of 1.00 were selected as influential ones and the corresponding chromatographic intensity (height) was considered. 

Table 1 shows the selected volatile variables and the corresponding retention time intervals for each vegetable oil matrix. 

### 3.2. Experimental Data Matrix of Ageing

As stated before, data were arranged in two different data matrices: (i) 252 × 22 olive-pomace oil data matrix; and (ii) 468 × 20 sunflower, grapeseed, rapeseed, corn, and blended oils data matrix.

Each row corresponds to each sample at a specific storage time and each column corresponds to the value of a single determined parameter. Table 2 shows all parameters or variables monitored for olive-pomace oil and a brief description of them. Regarding refined seed oils, peroxide value, K_270_, K_232_, ΔK, refractive index, oxidative stability, anisidine value, total tocopherols, α-tocopherol, β-tocopherol, γ-tocopherol, δ-tocopherol, volat1, volat2, volat3, volat4, and volat5 variables were considered. 

In addition, BHA, BHT, and PG variables, which refer to absolute content of each type of antioxidant, are given in mg/kg of vegetable oil. As the addition of preservatives is not allowed in olive-pomace oils, they were not tested for them.

### 3.3. Modelling the Ageing of Refined Olive-Pomace Oils

The experimental data matrix was autoscaled: variables were mean-centred and weighed by the standard deviation to give them equal variance. A multidimensional map of the 21 olive-pomace samples in relation to the 22 physicochemical parameters was obtained by PCA. Six PCs were selected that explain 73% of the total data variance. 

**Figure 1:**
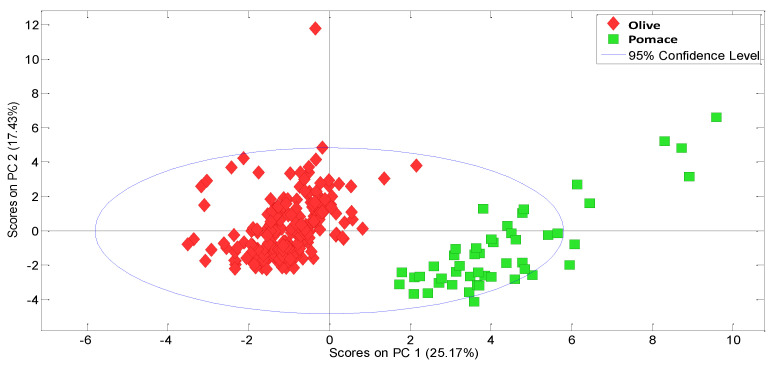
PC1–PC2 score plot obtained from the PCA model considering all variables for the olive-pomace oil.

**Figure 2:**
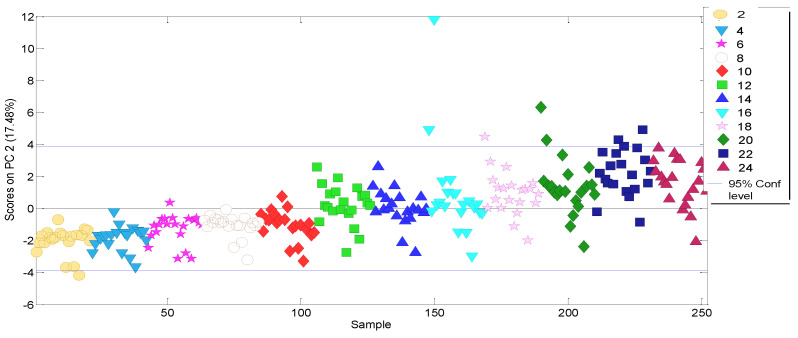
PC2 scores obtained from the PCA model considering all physicochemical selected variables for the olive-pomace oil. (Note that storage time is used only as marker in order to facilitate the understanding of natural behaviour over time).

The PC1–PC2 score plot, which explains the greatest variability in the data, shows a grouping of the samples according to the type of vegetable oil involved, discriminating between olive and pomace oil (Figure 1). However, based on the PC2 scores, a natural grouping of samples according to storage (ageing) time is evident (Figure 2).

No outliers were considered after checking the residual-leverage (Q-T^2^) screening plot.

#### 3.3.1. Selection of Significant Variables

A second PLS modelling was then applied to reveal possible relationships between storage time (y-variable) and the potentially significant dependent variables (x-variables). Four Latent variables (LVs) were then selected; LV1 explained 64% of the variance of the y-variable, which stated a high correlation between analytical parameters and storage time. 

**Figure 3:**
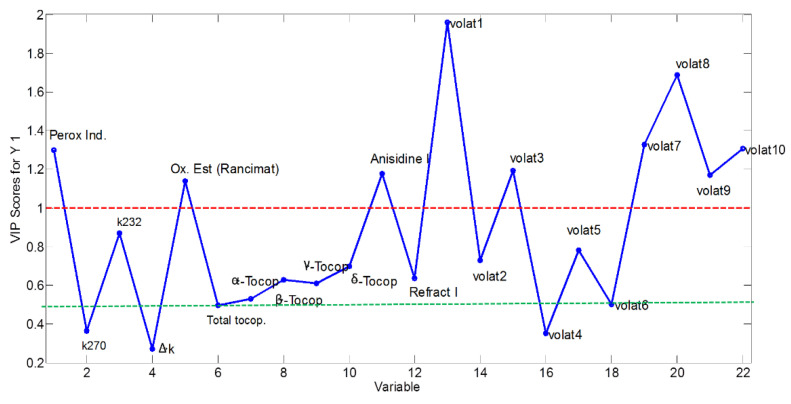
VIP scores profile obtained from the PLS model considering all variables.

The importance of the variables for the model is estimated by evaluating the loadings profile of the PLS model. For this purpose, the VIP coefficients are plotted against the variables (Figure 3). Based on previous experience, 0.5 was set as a threshold value: any variable with VIP below 0.5 was considered to be unrelated over time. Variables with VIP between 0.5 and 1 were considered ‘borderline’ and a Pearson correlation analysis of each variable with respect to storage time was then applied in order to verify the potential significance on the model. Pearson correlations between pairs of variables were also evaluated to avoid highly correlated variables which could cause overfitting of the model. Table 3 shows a summary of excluded and included variables in the multivariate models for the rest of the study.

#### 3.3.2. Kinetic Parameters 

**Figure 4:**
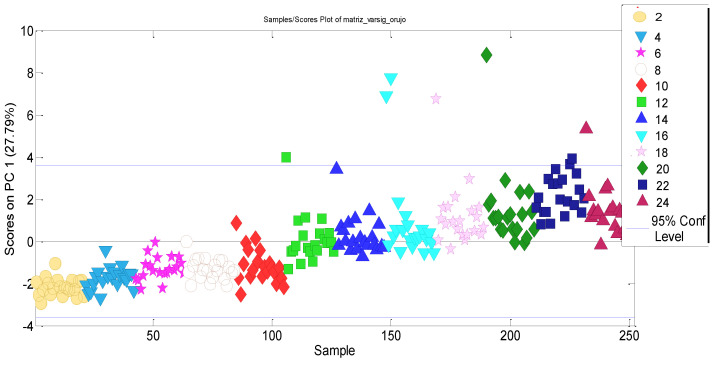
PC1 scores obtained from the PCA model considering only ageing-related variables for the olive-pomace oil. (Note that storage time is used only as a marker in order to facilitate the understanding of natural behaviour over time).

A new PCA model was built considering only the selected relevant variables (252 × 12 data matrix). This time, five PCs were selected, increasing both the total cumulative variance explained by the model (>72%) and the variance explained by PC1 (28%) with respect to the former model. Figure 4 clearly shows PC1 is now time-structured, making it suitable for estimating the kinetic parameters: samples were ordered from negative scores for PC1 in the youngest olive-pomace oils, to positive greater values when storage time increases. PC1–PC2 score plot no longer shows discrimination between olive and pomace oils.

Fitting a multivariate kinetic model involves the kinetic description of the important degradation reactions based on the PC1 scores, assuming that the degradation reactions are the main sources of variability in the data set. Robust bisquare fitting was applied. The best model fitting was obtained when the logarithms of PC1 scores were plotted vs. the storage times (coefficient of determination, R^2^ = 0.82, root mean square error, RMSE = 0.0506) so that a pseudo-first-order degradation kinetics could be regarded:

$$-\frac{\mathrm{dQ}}{\;\mathrm{dt}\;}=\;k\cdot Q\;;\;\ln Q\;={\;\mathrm{lnQ}}_{0}-k\cdot t$$

where Q is a multivariate parameter that gathers the PC1 scores of the samples as a function of time, Q_0_ is the score of the fresh sample, and k is the oxidation kinetic constant. Values of Q_0_ = 1.165 and k = 0.031 were calculated. 

#### 3.3.3. Shelf Life Modelling

**Figure 5:**
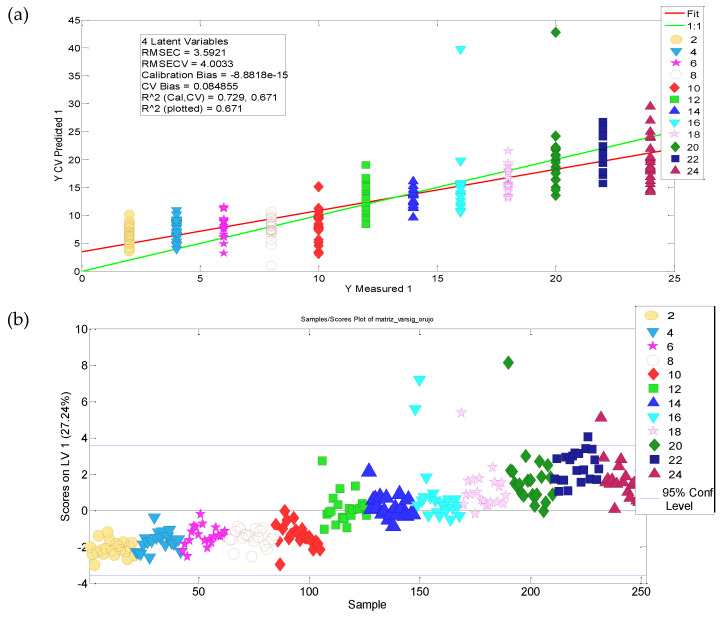
(**a**) Relationship between predicted and known values of the independent y-variable (ageing time) obtained from the PLS model considering the selected significant variables; (**b**) LV1 scores used to establish the shelf life model equation.

A last PLS regression model was set up considering only the relevant x-variables. Four LVs were selected, taking into account that LV1 explained more than 65% of the y-variance, i.e., storage time variance. Finally, the linear model between the model predicted and actual y-variable values proved to be suitable, showing a goodness of fit of 0.73 with an RMSE of 0.08 (Figure 5a). However, two trends in the data could be distinguished; samples from 2 to 10 months of ageing have similar scores. Thus, it seems that in these first months the vegetable oils almost did not evolve, and it is after 10 months of storage that the slope of the LV1 scores increases (see Figure 5b).

LV1 scores were used to establish a linear model (Q(t) = a + b·t) of autoscaled experimental data vs. storage time (R^2^ = 0.84; RMSE = 0.73). The estimated coefficients (95% confidence interval) were: a = 2.702 (−2.896, 2.509) and b = 0.1977 (0.1845, 0.2108). 

As argued previously, the acceptability limit vector collects the regulatory threshold values of the monitored physicochemical characteristics under study as well as robust statistics mean values of non-regulatory parameters at storage time in which the vegetable oils can no longer be considered as complying with the requirements. In this case, only PV is regulated. An olive-pomace oil sample should be considered unfit for consumption if the PV is 15 mEq/kg or more [20].

When comparing the evolution of the peroxide value median calculated from of all the samples with the regulatory value, it increases from 12.2 at 14 months of storage to 19 when samples had been stored for 16 months. Furthermore, the oxidative stability decreased from 18.8 to 13.5 h in month 14 of storage and then remained stable (taking into account the error of the method) until the end of the study. 

Therefore, the acceptability limit vector, Q_A_, is defined from the median values of each variable included in the fitted model considering all the samples at a storage time of 14 months, with the only exception of the peroxide value, which is set at 15 according to the current legislation. Table 4 shows the acceptability limit vector values. 

**Q_A_** vector was autoscaled and multiplied by **L**, the loading vector of LV1, which explains the largest variance of the y-variable (storage time), regarding the PLS shelf life model. A value of 0.155 was obtained for Q_C_, which was interpolated in the regression equation of the multivariate shelf life model to obtain a t_C_ value. Fourteen months were finally estimated to be a representative shelf life time for olive-pomace oils stored under the experimental conditions described in this study. 

#### 3.3.4. Peroxide Value-Based Modelling—Univariate Approach 

At this point the question arises as to what results we would have obtained based only on the empirical modelling of the PV data, in a univariate approach, taking into account that it is the only parameter whose value is regulated and has been taken into account to establish the limit vector. A conventional least-squares regression of the peroxide data with respect to ageing time was performed in order to obtain the (purely empirical) model that best fits and explains the variation of the PV over ageing time. The best fit of the data is achieved with the multiplicative form of the model (R^2^ = 0.67; mean absolute error = 0.23):

PV = a · t ^b^

Observations with residuals greater than 3 were considered outliers and were removed from the model (five outliers). The equation of the fitted (linearised) model was:

ln PV = 1.021 + 0.605 · ln t

From this model, the shelf life time was calculated to be 16 months when the PV is interpolated to 15 mEq/kg. This one result is an overestimation compared to the value estimated with the multivariate approach. 

### 3.4. Modelling the Ageing of Refined Seed Oils 

**Figure 6:**
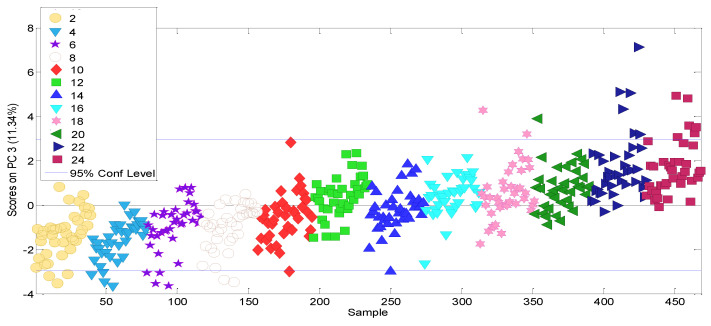
PC3 scores for the refined seed oils PCA modelling. (Note that storage time is used only as a marker in order to facilitate the understanding of natural behaviour over time).

As already explained, a PCA model is now built considering all the variables under study. Six PCs, explaining the 65% of the total data variance, were selected. The PC1–PC2 scores plot shows a sample grouping according to the type of vegetable oil involved (notice that blended seed oils appear with the same scores as sunflower oil, which could be because the sunflower oil is the major vegetable oil in the blend). PC3 has the highest scores with regard the increase in storage time (see Figure 6). No outliers were considered. 

#### 3.4.1. Selection of Significant Variables

VIPs-based PLS modelling and between-variables Pearson’s correlations were evaluated. Significant selected variables were reduced to the following: PV; K_270_; oxidative stability; total tocopherols; β-tocopherol; anisidine value; volat1; volat2; volat3; and volat5. 

#### 3.4.2. Kinetic Parameters 

**Figure 7:**
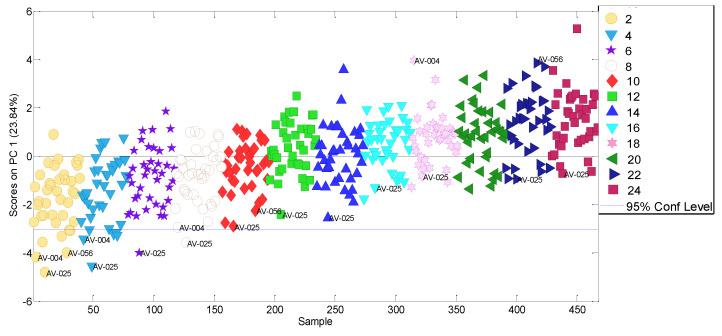
PC1 scores obtained from the PCA model considering only ageing-related variables for the refined seed oil.

A PCA model considering only the relevant x-variables (468 × 10 data matrix) was built. Six PCs were selected which showed an increase in both the total model-explained cumulative variance (>80%) and the PC1-expained variance (24%) with respect to the former model. This time, the PC1–PC2 scores plot no longer shows grouping by vegetable oil type as before. PC1 is time-structured and therefore suitable for estimating the kinetic parameters. It was also observed that three samples have lower PC1 scores than expected, particularly within the first months (see Figure 7). These samples all are high oleic sunflower oils, so it could be thought that their degradation kinetics are lower than the remaining samples at the beginning of the storage time, while as the oxidation progresses these differences are not so clear. 

**Figure 8:**
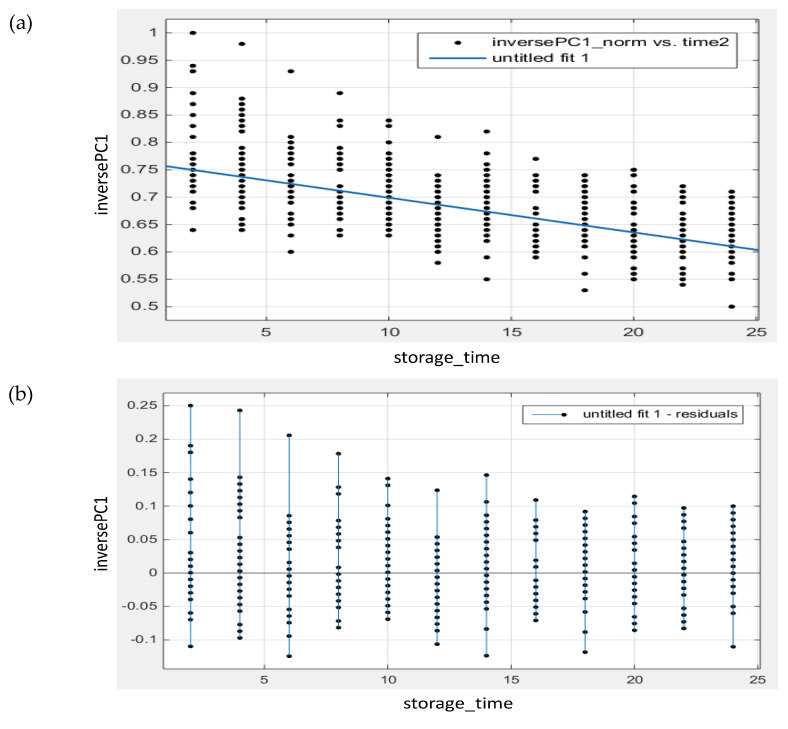
(**a**) Relationship between the inverse of the PC1 scores and storage time (multivariate kinetic modelling); (**b**) Residual plot for the kinetic model.

A multivariate kinetic model with PC1 scores was fitted. The best results were obtained when fitting a pseudo-second-order degradation kinetics equation (R^2^ = 0.4437, RMSE = 0.0559):

$$-\frac{\mathrm{dQ}}{\;\mathrm{dt}\;}=\;k\cdot {\left[Q\right]}^{2}\;;\;\frac{1}{Q}=\frac{1}{Q_{0}}+2k\cdot t$$

where Q collects the PC1 scores of the samples as a function of the storage time, Q_0_ is the score of the fresh sample, and k is the oxidation kinetic constant. Values of Q_0_ = 1.312 and k = −0.0032 were calculated. In this case, the fit to the model was unsatisfying. During the first months of ageing, a large data scattering can be observed, and thus high residual values, although these differences were not as pronounced at the end times of the study (see Figure 8). This could be a consequence of the high variability of the vegetable oil samples and thus the discrepancies in the oxidation rates. 

#### 3.4.3. Shelf Life Modelling 

A supervised PLS regression model was set up considering only the relevant x-variables. Four LVs were selected, taking into account that LV1 explained almost 60% of the y-variance, i.e., storage time variance. A goodness of fit of 0.63 with an RMSE of 0.05 was obtained when y-predicted vs. y-measured is plotted. LV1 scores were used to establish a linear model (Q(t) = a + b·t) between autoscaled experimental data and storage time (R^2^ = 0.67; RMSE = 0.87). The estimated coefficients (95% confidence interval) were: a = −2.135 (−2.304, −1.965) and b = 0.1602 (0.1487, 0.1717). 

Similarly, only a PV less than 10 mEq/kg is required for a refined seed oil to be considered as meeting the mandatory requirements of its category [21]. The median of all the peroxide values exceeds the regulatory value when samples had been ageing for 10 months. Therefore, the acceptability limit vector, **Q_A_**, is defined considering the median values of each variable included in the model at month 10, except for the PV which is set at 10 mEq/kg to be compliant with the current regulation. Table 5 shows the acceptability limit vector values. 

**Q_A_** vector was autoscaled and multiplied by **L**, the loading vector of LV1 corresponding to the PLS shelf life model. A value of −0.625 was obtained for the Q_C_ scalar. This estimated value was interpolated into the regression equation of the multivariate PLS shelf life model to obtain a representative t_C_ value. Nine months were finally estimated to be a suitable shelf life time for refined seed oils stored under the experimental conditions described in this study. 

### 3.5. Shelf Life Index and Ageing Rate 

Once the shelf life model is established, and t_C_ is estimated, three indexes related to stability and compliance with requirements are calculated: equivalent ageing time, shelf life index, and ageing rate. 

Equivalent ageing time, t_i_, is extrapolated from the shelf life model equation by calculating Q_i_:

Q_i_ = **X_i_** · **L**

where **X_i_** symbolizes the autoscaled vector of the experimental data of each sample at a specific time, and **L** is the vector of loadings of the first latent variable, LV1, from the PLS model. 

The shelf life index, I_SL_, reflecting the number of months an oil continues in compliance with the requirements of its category, is defined as:

$$I_{\mathrm{SL}}={\;t}_{C}-t_{i}$$

And finally ageing rate, %Age, calculated as follows:

$$\%\mathrm{Age}\;=\;\frac{t_{i}}{{\;t}_{C\;}}\cdot 100$$

Similarly, these indices are calculated for the univariate PV-based approach in order to compare the results obtained by both approaches. 

#### 3.5.1. Comparison of Results (Multivariate and Univariate Approaches) 

Table 6 shows the index results obtained for five olive-pomace samples randomly selected from among those included in the study. 

Considering only the PV, vegetable oil AV002 would no longer comply with the standard as of month 8 of ageing. However, the multivariate model predicts that this vegetable oil is compliant until month 10. The opposite applies to AV019. The multivariate model detects that this vegetable oil is suitable only up to month 14. Considering the PV-based empirical model, the vegetable oil could remain acceptable for 3.5 months beyond month 14. A similar behaviour is predicted for the AV026 vegetable oil. 

AV030 is a vegetable oil that has aged for 14 months before being subjected to analytical controls. The multivariate model detects that the oil is already oxidised and close to the limit of acceptability. However, the PV-based univariate model predicts that it would remain compliant for another seven months. 

Finally, AV031 would no longer be compliant from month 6 onwards considering only the PV. On the contrary, the multivariate model showed that sample is suitable until month 12 of storage, when the analytical values exceed the acceptability limits. 

#### 3.5.2. Verifying the Shelf Life Prediction Capability (Multivariate and Univariate Approaches)

The predicted lifetime from the multivariate shelf life model, taking as a reference the state of each of the oils at month 2, is then compared with the actual date on which the olive pomace oil does not meet the required quality parameters. Note that a two-month uncertainty in the predictions is taken into account; this corresponds to the time that elapses from two consecutive analytical controls (experimental work frequency). 

In all cases of study, the shelf life indexes at month 2 predict the number of months for each vegetable oil to be consistent with requirements. As an example, AV002 is predicted to be suitable for an additional 10 months, and after only 10 months of ageing it has just exceeded the acceptability limit of oxidative-related quality parameters. AV031 is predicted to keep 8 months of shelf life, while in month 10 of ageing it presents a shelf life index of 1, about to be considered unsuitable. On the contrary, the empirical PV-based model does not perform successful predictions of the suitability of vegetable oils. AV002 is predicted to conserve 15 months before reaching the end of its shelf life based on the prediction after two months of storing. However, already in month 8 of ageing it presents an ageing rate above 100%, which is not in compliance with the quality parameters corresponding to its category (peroxide value 15.8 mEq/kg). 

A similar prediction result comparison between univariate and multivariate seed oil models is missing. As stated before, degradation model fitting is not as good, showing a large dispersion of data supposing different rates of degradation of considered samples. Any prediction of oxidation times from a model that does not fit the reality of the data will be biased. 

## 4. Conclusions

The shelf life of refined edible vegetable oils has been evaluated by considering the changes they undergo as a whole, and applying a suitable multivariate approach. Refined vegetable oils obtained from the olive fruit such as pomace and olive oils (note that the latter name refers to a marketed blend of virgin and refined olive oil) showed a longer shelf life time than seed-obtained oils. These results are consistent with the findings of other stability comparison studies found in the literature [25]. The established shelf life regression models are an easy, simple, and good tool to assess the actual oxidation status and time remaining in compliance with the accepted requirements for each refined vegetable oil sample. The predictability of the multivariate approach has been proved satisfactory as compared to an empirical model related only to PV. Discrepancies were found in the oxidation rates of oils extracted from different plant seeds, particularly during the first months of ageing. Unfortunately, it is not possible to compare the shelf life prediction with other studies or papers. Most of the available studies are based on comparisons of stability without actually calculating a finite period of useful lifetime due to a lack of definition of an adequate acceptability limit vector. To our knowledge, neither stability nor shelf life in previous studies of refined oil under real-time oxidation conditions has been carried out. This study is an example of the potential of chemofoodmetrics for solving food quality problems. Future research will be carried out by increasing the number of samples of each seed type and modelling analytical data separately, to obtain a better model fitting for the purpose. 

## Figures and Tables

**Table 1:** Influential volatile variables related to the volatile chromatographic fingerprint based on the storage time.

(i) Olive-Pomace Oil Matrix			(ii) Seed Oil Matrix		
Name	Variable Number	Retention Time Interval (min)	Name	Variable Number	Retention Time Interval (min)
Volat1	77	1.60–1.68	Volat1	76	1.58–1.69
Volat2	167	1.77–1.79	Volat2	196	1.82–1.84
Volat3	195	1.81–1.84	Volat3	521	2.34–2.48
Volat4	337	2.04–2.12	Volat4	846	2.89–2.93
Volat5	491	2.31–2.32	Volat5	871	2.93–3.01
Volat6	518	2.33–2.47			
Volat7	845	2.90–2.92			
Volat8	869	2.93–2.97			
Volat9	2092	4.96–5.01			
Volat10	2124	5.03–5.06			

**Table 2:** Physicochemical and sensory parameters being monitored in olive-pomace oil study.

No	Parameter	Brief Description
1.	Peroxide value	Peroxide content expressed in terms of milliequivalents of active oxygen per kilogram of vegetable oil.
2.	K_270_	The specific absorbances are calculated for a concentration of 1% (m/V) in a 10 mm cell (absorbance units × (g/100 mL)^–1^ × cm^–1^).
3.	K_232_	
4.	ΔK	
5.	Refractive index	At 20 °C as reference temperature (no units).
6.	Oxidative stability	Rancimat induction period (in hours) at 120 °C.
7.	Anisidine value	Rate of increase of absorbance, at 350 nm in a 10 mm cell, when reacted with p-anisidine under specific conditions (no units).
8.	Total tocopherols	Absolute content of each type of tocopherol expressed in milligrams per kilogram of vegetable oil, determined by liquid chromatography.
9.	α-tocopherol	
10.	β-tocopherol	
11.	γ-tocopherol	
12.	δ-tocopherol	
13.	Volat1	Normalized chromatographic intensities (heights) at specific retention time values previously selected, extracted from the corresponding volatile chromatographic fingerprint (no units).
14.	Volat2	
15.	Volat3	
16.	Volat4	
17.	Volat5	
18.	Volat6	
19.	Volat7	
20.	Volat8	
21.	Volat9	
22.	Volat10	

**Table 3:** Summary of both selected and excluded variables on the olive-pomace multivariate shelf life model.

Selected Variables		Excluded Variables	
Peroxide value	Volat1	K_270_	Volat4
Oxidative stability	Volat2	K_232_ *	Volat6
Anisidine value	Volat3	ΔK	Volat10 *
α-Tocopherol	Volat5	Total tocopherols	
γ-Tocopherol	Volat7	β-Tocopherol *	
	Volat8	δ-Tocopherol	
	Volat9	Refractive index *	

* Excluded variables for having correlation coefficients greater than 0.7 with any of the selected variables.

**Table 4:** Acceptability limit values used for building the scores vector to be applied to the olive-pomace oil shelf life model.

Variable	Acceptability Limit	Variable	Acceptability Limit
Peroxide value	15.0	Volat1	0.196
Oxidative stability	13.4	Volat2	0.143
Anisidine value	5.4	Volat3	0.130
α-Tocopherol	174.7	Volat5	0.086
γ-Tocopherol	10.3	Volat7	0.108
		Volat8	0.084
		Volat9	0.073

**Table 5:** Acceptability limit values used for building the scores vector to be applied to the refined seed oil shelf life model.

Variable	Acceptability Limit	Variable	Acceptability Limit
Peroxide value	10.00	Anisidine value	4.65
K_270_	4.24	Volat1	0.125
Oxidative stability	4.50	Volat2	0.023
Total tocopherols	665.11	Volat3	0.789
β-Tocopherol	28.66	Volat5	0.044

**Table 6:** Equivalent ageing time, t_i_, shelf-life index, I_SL_, and ageing rate, %Age, estimated values for five randomly selected refined olive or pomace oils to be used as representative samples in order to assess the reliability of the predictions.

		Multivariate Approach PLS (LV1 Scores) Model			Univariate ApproachEmpirical (Peroxide Value) Model			
Sample	Ageing Time	t_i_	I_SL_	%Age	PV	t_i_	I_SL_	%Age
AV002	2	3.7	10.3	26	2.5	0.8	15.2	5
	4	2.5	11.5	18	3.8	1.7	14.3	11
	6	3.5	10.5	25	6.2	3.8	12.2	24
	8	10.0	4.0	72	15.8	17.7	−1.7 *	111 *
	10	14.3	−0.3 *	102 *	26.7	42.1	−26.1 *	263 *
AV019	2	2.0	12.0	15	5.1	2.7	13.3	17
	4	4.0	10.0	28	7.0	4.6	11.4	29
	6	5.5	8.5	39	8	5.7	10.3	36
	8	7.7	6.3	55	8.8	6.7	9.3	42
	10	8.3	5.7	59	9.1	7.1	8.9	44
	12	12.2	1.8	87	9.2	7.2	8.8	45
	14	16.6	−2.6 *	118 *	12.8	12.5	3.5	78
AV026	2	3.3	10.7	24	2	0.6	15.4	4
	4	5.8	8.2	41	12.0	11.2	4.8	70
	6	10.6	4.4	75	8	5.7	10.3	36
	8	9.2	4.8	66	10	8.3	7.7	52
	10	11.4	2.6	81	10.5	9	7.0	56
	12	18.2	−4.3 *	130 *	10.5	9	7.0	56
	14	18.0	−4.0 *	129 *	9.4	7.5	8.5	47
	16	23.0	−9.0 *	164 *	12	11.2	4.8	70
AV030	14	13.7	0.3	98	10.5	9	7.0	56
	16	16.9	−2.9 *	121 *	20	26.1	−10.1 *	163 *
	24	19.6	−5.6 *	140 *	12.9	12.7	3.3	79
AV031	2	8.1	5.9	58	10	8.3	7.7	52
	4	11.5	2.5	82	14	14.5	1.5	91
	6	13.1	0.9	94	15	16.2	−0.2 *	101 *
	8	11.4	2.6	81	16.3	18.6	−2.6 *	116 *
	10	13.0	1.0	93	19.4	24.8	−8.8 *	155 *

Values in italics * indicate that the vegetable oil under consideration is not suitable for consumption.

## Data Availability

The data presented in this study are available on request from the corresponding author. The data are not publicly available due to confidentiality.

## Supplementary Materials

The following supporting information can be downloaded at: https://www.mdpi.com/article/10.3390/molecules27196508/s1, Table S1: Raw data obtained in the fatty acid profile analysis by GC-FID chromatography at the beginning of the study for all the oil samples.

## Sample Availability

Samples of the vegetable refined oils are available from the authors.
