# Supplementary material for: Monitoring the Shelf Life of Refined Vegetable Oils under Market Storage Conditions—A Kinetic Chemofoodmetric Approach
Source: Molecules. 2022 Oct 2;27(19):6508. doi: 10.3390/molecules27196508 (PMC9573321; doi:10.3390/molecules27196508)
Supplement: Supplementary file 1 [file molecules-27-06508-s001.zip › molecules-1892619-supplementary.pdf]

Table S1. Raw data obtained in the fatty acid profile analysis by GC-FID chromatography at the beginning of the study for all the oil samples.

S- sunflower oil

G- grapeseed oil

R- rapeseed oil

C- corn oil

B- Blend oil

P- pomace oil

O- Olive oil

Code	Lauric	Miristic	Palmitic	Palmitoleic	Margaric	Margaroleic	Esteáric	Oleic	Linoleic	Ararquic	Linolenic	Gadoleic	Behenic	Erucic	C22:2	Lignoceric	Nervonic
S-003	0.00	0.07	5.93	0.11	0.04	0.03	3.22	33.44	55.71	0.24	0.06	0.15	0.73	0.00	0.00	0.27	0.00
S-004	0.00	0.04	4.09	0.15	0.03	0.04	3.17	80.03	10.45	0.31	0.05	0.27	0.97	0.01	0.01	0.36	0.01
S-009	0.00	0.07	6.45	0.14	0.04	0.03	3.84	27.56	60.35	0.29	0.11	0.13	0.71	0.00	0.00	0.28	0.01
S-017	0.00	0.06	5.84	0.13	0.03	0.04	3.38	33.44	55.56	0.26	0.06	0.15	0.75	0.00	0.00	0.29	0.01

S-021	0.00	0.07	6.01	0.12	0.03	0.03	3.60	30.08	58.53	0.28	0.06	0.15	0.74	0.00	0.00	0.31	0.00
S-024	0.00	0.06	5.80	0.12	0.04	0.03	3.64	33.56	55.08	0.29	0.11	0.18	0.71	0.00	0.00	0.37	0.01
S-025	0.00	0.04	4.00	0.16	0.03	0.04	2.99	82.48	8.35	0.29	0.05	0.27	0.92	0.01	0.00	0.35	0.01
S-027	0.00	0.06	5.90	0.10	0.03	0.03	3.38	31.08	57.83	0.26	0.09	0.16	0.77	0.00	0.00	0.31	0.00
S-029	0.00	0.07	6.10	0.13	0.04	0.03	3.62	29.37	59.02	0.28	0.07	0.18	0.76	0.00	0.00	0.33	0.00
S-039	0.00	0.07	6.04	0.13	0.03	0.03	3.39	31.36	57.41	0.27	0.06	0.15	0.73	0.00	0.00	0.32	0.00
S-040	0.00	0.07	6.32	0.14	0.03	0.03	3.23	30.61	57.96	0.27	0.06	0.16	0.75	0.00	0.00	0.36	0.00
S-041	0.00	0.06	5.99	0.13	0.03	0.03	3.27	32.65	56.23	0.26	0.06	0.17	0.75	0.00	0.00	0.34	0.01
S-042	0.00	0.05	5.00	0.17	0.03	0.04	3.07	65.42	24.34	0.29	0.05	0.24	0.89	0.01	0.00	0.40	0.01
S-043	0.00	0.07	6.40	0.14	0.03	0.03	3.21	30.41	58.08	0.26	0.11	0.16	0.77	0.01	0.00	0.32	0.00
S-045	0.00	0.07	6.25	0.15	0.03	0.03	3.20	31.94	56.82	0.25	0.07	0.15	0.69	0.00	0.00	0.33	0.00
S-049	0.00	0.07	6.71	0.16	0.04	0.03	3.84	28.44	59.14	0.29	0.08	0.14	0.72	0.01	0.00	0.32	0.01
S-050	0.00	0.07	6.21	0.14	0.03	0.03	3.11	30.39	58.46	0.25	0.07	0.17	0.71	0.00	0.00	0.36	0.01
S-051	0.00	0.08	6.79	0.17	0.03	0.03	3.05	30.66	57.62	0.26	0.06	0.18	0.69	0.00	0.00	0.37	0.00
S-053	0.00	0.06	6.28	0.12	0.04	0.03	3.64	30.90	57.26	0.29	0.07	0.15	0.82	0.00	0.00	0.35	0.00
S-054	0.00	0.07	6.20	0.13	0.03	0.03	3.13	30.41	58.45	0.25	0.06	0.15	0.71	0.00	0.00	0.36	0.00
S-055	0.00	0.07	6.10	0.13	0.03	0.03	3.18	30.70	58.14	0.26	0.07	0.18	0.74	0.00	0.00	0.37	0.00
S-	0.00	0.04	4.39	0.17	0.03	0.05	3.20	76.98	13.16	0.33	0.11	0.24	0.92	0.01	0.00	0.35	0.01

056																	
S-057	0.00	0.06	5.84	0.10	0.03	0.03	3.31	32.93	56.09	0.26	0.12	0.16	0.76	0.01	0.00	0.30	0.01
S-060	0.00	0.07	5.83	0.13	0.03	0.03	3.17	34.30	54.82	0.25	0.06	0.16	0.73	0.00	0.00	0.42	0.00
S-061	0.00	0.06	5.74	0.12	0.03	0.03	3.00	34.24	54.48	0.25	0.07	0.17	0.72	0.18	0.00	0.62	0.29
S-070	0.00	0.05	7.51	0.17	0.00	0.00	3.68	26.38	61.29	0.18	0.06	0.06	0.46	0.00	0.00	0.16	0.00
S-071	0.00	0.08	6.71	0.16	0.03	0.03	3.20	31.84	56.32	0.27	0.06	0.16	0.73	0.00	0.00	0.39	0.00
S-072	0.00	0.07	6.12	0.12	0.03	0.03	3.34	31.43	57.34	0.26	0.10	0.16	0.72	0.00	0.00	0.28	0.00
S-073	0.00	0.08	6.08	0.13	0.04	0.04	3.41	31.53	57.19	0.27	0.09	0.16	0.72	0.00	0.00	0.28	0.00
G-022	0.00	0.04	6.85	0.13	0.06	0.03	4.37	20.02	67.65	0.21	0.36	0.17	0.05	0.00	0.00	0.05	0.00
R-023	0.01	0.04	4.47	0.22	0.05	0.06	1.83	60.38	23.31	0.56	7.29	1.10	0.36	0.05	0.00	0.14	0.12
C-052	0.00	0.03	11.00	0.13	0.06	0.03	2.09	30.50	54.07	0.51	0.88	0.27	0.20	0.01	0.00	0.22	0.00
C-058	0.00	0.03	10.68	0.13	0.06	0.03	1.96	31.04	54.04	0.49	0.87	0.27	0.17	0.01	0.00	0.21	0.00
C-059	0.00	0.03	10.65	0.13	0.06	0.03	2.02	30.46	54.38	0.49	1.03	0.28	0.19	0.01	0.00	0.21	0.00
C-067	0.00	0.03	11.41	0.16	0.07	0.04	1.93	30.74	53.44	0.51	0.97	0.28	0.17	0.00	0.00	0.24	0.00
B-001	0.00	0.06	5.15	0.16	0.04	0.04	3.05	51.72	36.30	0.33	1.67	0.41	0.73	0.05	0.00	0.26	0.03
B-047	0.42	0.22	7.50	0.50	0.05	0.08	2.93	69.42	16.98	0.39	0.30	0.29	0.63	0.01	0.00	0.28	0.00
B-048	0.00	0.07	6.21	0.16	0.03	0.04	3.22	36.10	52.23	0.29	0.34	0.24	0.68	0.01	0.00	0.35	0.01
B-068	0.01	0.07	6.90	0.12	0.04	0.03	3.12	32.72	54.47	0.26	1.17	0.17	0.64	0.00	0.00	0.28	0.00

O-013	0.00	0.01	12.24	1.21	0.06	0.10	3.36	73.46	8.02	0.44	0.67	0.23	0.12	0.00	0.00	0.07	0.00
O-018	0.00	0.01	11.86	1.11	0.09	0.16	2.77	75.29	7.09	0.45	0.71	0.28	0.12	0.00	0.00	0.06	0.00
O-019	0.00	0.01	13.51	1.45	0.07	0.11	3.08	69.49	10.69	0.47	0.69	0.23	0.13	0.00	0.00	0.07	0.00
O-020	0.00	0.01	12.51	0.95	0.08	0.13	3.40	73.70	7.60	0.45	0.68	0.25	0.14	0.00	0.00	0.07	0.00
O-026	0.00	0.01	11.03	0.89	0.08	0.12	3.09	76.00	7.17	0.44	0.68	0.29	0.13	0.00	0.01	0.06	0.00
O-028	0.00	0.01	10.26	0.77	0.07	0.10	3.46	79.07	4.85	0.40	0.62	0.24	0.10	0.00	0.00	0.05	0.00
O-030	0.00	0.01	11.12	0.96	0.08	0.16	2.80	75.74	7.47	0.46	0.68	0.30	0.14	0.00	0.00	0.07	0.00
O-031	0.00	0.01	11.36	1.00	0.06	0.11	3.38	75.02	7.43	0.43	0.71	0.26	0.15	0.00	0.00	0.07	0.00
O-032	0.00	0.01	11.95	1.06	0.08	0.13	3.19	73.45	8.50	0.46	0.67	0.26	0.16	0.00	0.00	0.08	0.00
O-036	0.00	0.01	11.27	0.96	0.06	0.11	3.08	76.05	6.83	0.45	0.70	0.27	0.14	0.00	0.00	0.07	0.00
O-063	0.00	0.01	12.05	1.21	0.08	0.12	3.28	71.67	9.96	0.48	0.67	0.25	0.14	0.00	0.00	0.08	0.00
O-064	0.00	0.01	12.03	1.17	0.06	0.11	3.39	73.61	8.02	0.45	0.69	0.24	0.14	0.00	0.00	0.07	0.00
O-066	0.00	0.02	13.33	1.38	0.09	0.14	3.04	69.82	10.53	0.48	0.68	0.25	0.15	0.00	0.00	0.10	0.00
O-075	0.00	0.02	10.90	0.85	0.07	0.11	3.21	77.23	6.10	0.41	0.64	0.25	0.15	0.00	0.00	0.07	0.00
O-076	0.00	0.01	13.11	1.40	0.08	0.13	3.05	70.42	10.18	0.46	0.70	0.25	0.14	0.00	0.00	0.08	0.00
O-077	0.00	0.01	12.07	1.19	0.07	0.11	3.24	72.23	9.49	0.45	0.70	0.25	0.12	0.00	0.00	0.07	0.00
O-078	0.00	0.01	12.88	1.29	0.07	0.13	2.98	70.87	10.15	0.47	0.67	0.26	0.14	0.00	0.00	0.08	0.00
O-	0.00	0.01	11.29	1.00	0.06	0.10	3.37	75.31	7.34	0.45	0.63	0.25	0.12	0.00	0.00	0.07	0.00

079																	
P-002	0.00	0.02	11.95	1.02	0.08	0.14	2.99	71.01	10.84	0.49	0.76	0.36	0.22	0.01	0.00	0.11	0.00
P-038	0.00	0.02	11.42	0.97	0.07	0.11	2.86	70.31	12.25	0.50	0.81	0.37	0.20	0.01	0.00	0.11	0.00
P-065	0.00	0.02	10.83	0.86	0.07	0.11	2.81	71.96	11.36	0.49	0.82	0.36	0.20	0.00	0.01	0.10	0.00
P-074	0.00	0.01	11.84	1.02	0.08	0.14	2.86	71.32	10.85	0.49	0.72	0.33	0.22	0.00	0.00	0.11	0.00
